# Subacute Paget–Schroetter with rapid recurrence and concurrent pulmonary emboli: a case report

**DOI:** 10.3389/fsurg.2025.1705410

**Published:** 2025-11-20

**Authors:** Francis O’Toole, Shantele Kemp Van Ee, Danielle Madison, Kathryn DiVincenzo, Khoa Tran, Alaa Mohamed

**Affiliations:** 1 Nova Southeastern University Dr. Kiran C. Patel College of Osteopathic Medicine, Davie, FL, United States; 2 Emergency Department, Graduate Medical Education, Lakeland Regional Health Medical Center, Lakeland, FL, United States; 3 Internal Medicine, Graduate Medical Education, Lakeland Regional Health Medical Center, Lakeland, FL, United States

**Keywords:** Paget–Schroetter, pulmonary embolus, effort thrombosis, case report, thrombectomy, first rib resection and scalenectomy, catheter directed thrombolysis (CDT)

## Abstract

Effort thrombosis, also known as Paget–Schroetter syndrome, is a rare venous manifestation of Thoracic Outlet Syndrome, primarily affecting young adults who perform repetitive overhead motions, such as athletes and trades workers. Stasis occurs when surrounding tissues encroach upon venous flow through the subclavian and/or axillary veins, manifesting as pain, swelling, and discoloration, with a potential for pulmonary embolism and other sequelae if left untreated. We present a 22-year-old male, avid weightlifter, with initial and recurrent left subclavian thrombosis and right pulmonary emboli, who did not respond to mechanical thrombectomy and anticoagulation, requiring surgical reconstruction at the thoracic outlet only two weeks after initial presentation. While there are no consensus guidelines for the management of Paget–Schroetter syndrome, aggressive management of acute thrombi demonstrates low rates of recurrence. There is less guidance regarding subacute thrombi. We advocate for a similarly aggressive approach to management of subacute thrombi to prevent recurrence and complications such as pulmonary embolism, post-thrombotic syndrome, and vessel fibrosis.

## Introduction

Paget–Schroetter syndrome (PSS) is a condition affecting the upper extremities characterized by the formation of a deep vein thrombus (DVT) in the subclavian or axillary veins (1). It manifests with symptoms of swelling, hyperemia, and pain in the affected upper extremity. Cases of this syndrome have been documented throughout the past 200 years, with the first detailed description in 1875 by James Paget (2). In 1884, Leopold von Schrötter was the first to propose vascular trauma from repetitive overhead activity as a potential etiology for the syndrome (3). It is uncommon, affecting fewer than 0.5–1 in 100,000 individuals annually (4). Those most affected by PSS are athletes or manual laborers who perform repetitive overhead activities.

Upper extremity DVT (UEDVT) can be categorized as either primary (idiopathic) or secondary. According to the 2012 CHEST guidelines for antithrombotic therapy for venous thromboembolic disease, PSS has been classically characterized as a primary etiology of UEDVT, accounting for more than 50% of all primary cases (5). More recently, it has been proposed that anatomic compression at the costoclavicular junction (CCJ) is a truly extrinsic factor contributing to the syndrome, rendering it a secondary cause (6). The other secondary causes of UEDVT are iatrogenic (catheter/pacemaker), malignancy, and hypercoagulability (6).

It has been estimated that primary etiologies account for approximately 33% of all UEDVT (6). Many cases initially diagnosed as primary UEDVT are often later found to have malignancy (5%–25% of cases) or a clotting abnormality (25%–42%) (6). Removing PSS and other later-discovered secondary causes, it can be realized that true, “primary” UEDVT is very rare, if it exists at all (6). Therefore, diagnosis and management should focus on identifying and empirically managing secondary causes, with a specific effort-related thrombosis and hypercoagulability workup in the absence of iatrogenic and neoplastic risk factors and suspicion.

**Figure 1:**
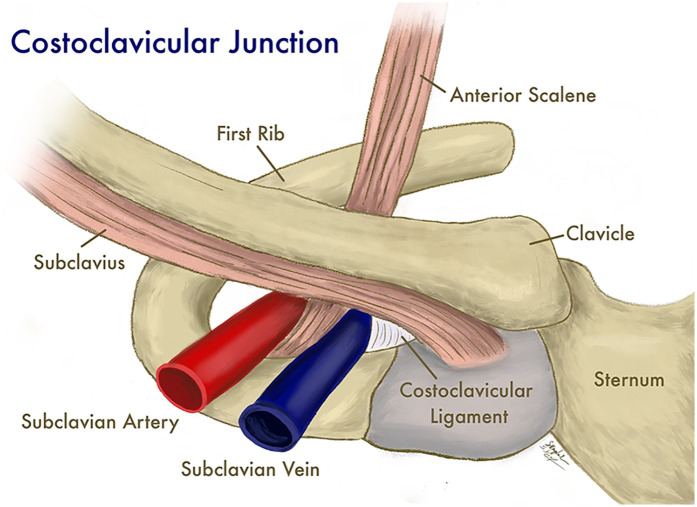
The subclavian vein and surrounding structures at the costoclavicular junction. (Illustration by Shantele and Sabrina Kemp Van Ee, used by permission).

Since activity should increase blood drainage from upper extremities and decrease risk of thrombosis, the relationship between a prothrombotic state and effort may initially seem counterintuitive. However, it has been proposed that the mechanism of venous stasis and a prothrombotic state is both anatomic and physiologic. In athletes, hypertrophy of the overhead musculature, particularly the anterior scalene, can occur, which may compress the subclavian vein as it passes through the thoracic inlet (1). This compression can be particularly evident in anatomic variants, where the costoclavicular ligaments may extend more laterally, contributing to the narrowing of the space through which the subclavian vein passes (Figure 1).

While anatomic compression may contribute to stasis of flow through the CCJ, it has also been proposed that repetitive microtrauma occurs to the subclavian vein, evidenced by the dense external fibrosis and adhesions commonly present around the vein during first rib resection (7). This microtrauma is also theorized to induce intimal hypertrophy, further contributing to the stasis of venous flow, with multiple cycles of partial thrombosis and recanalization occurring prior to the clinical manifestation of the syndrome (7). Unrealized thrombophilic states contribute to the formation of these thromboses in a large percentage of primary UEDVTs, but only in a minority of effort-related cases (6).

While the gold standard for diagnosing UEDVT is venography, compression ultrasonography has an excellent sensitivity of 97% and specificity of 96% (8). However, it is noted that the lack of compressibility in more proximal structures hinders this ultrasonographic method as a means of ruling out UEDVT (8). While neither diagnostic for UEDVT nor routinely recommended, a thrombophilic workup is prudent, as it may help identify the etiology of UEDVT and better inform management. A thrombophilic workup generally includes testing for Factor V Leiden mutation, prothrombin G20210A mutation, beta-2-glycoprotein, antiphospholipid antibodies, cardiolipin antibodies, antithrombin III titer, and protein C and S concentrations (9).

Most patients with PSS are managed with a combination of three components: oral anticoagulation, endovascular thrombolysis or thrombectomy, and surgical reconstruction. The specific combination of these per patient depends on the patient's age, functional status, and the age of the thrombus. Acute thromboses are generally considered to be less than 14 days old, subacute from 14 days to three months, and chronic older than three months (6). Treatment of acute thromboses is generally more structured, involving anticoagulation and catheter-directed thrombolysis, with recommended aggressive first rib resection as soon as one day after CDT (6). Subacute and chronic thromboses are more challenging and are managed on an individualized basis (6).

The principles of subacute treatment must consider an older and more organized clot, which is somewhat resistant to thrombolysis degradation. As such, CDT is generally not recommended beyond the acute period (6). The techniques available for managing younger subacute thrombi rely on anticoagulation to prevent pulmonary embolism, endovascular or surgical decompression of the thrombus, and correction of anatomic compression, if applicable (6). Young patients typically fall under the category of anatomic compression correction. The gold standard and definitive treatment for PSS compression is surgical resection of the first rib and anterior scalene, with or without the middle scalene (6).

While in the past PSS has been treated conservatively with simple limb elevation and anticoagulation alone, accepted management departed from this standard due to residual symptoms, disability, post-thrombotic syndrome, and recurrent thrombosis (1). Historically, less consideration has been given to PE in UEDVT compared to LEDVT, as it was thought to carry a lower incidence; however, there is at least mixed evidence regarding which location is most likely to be followed by PE (10). While a large study by Cote et al. found fewer UEDVTs with PE on presentation, the odds of death were higher in patients with non-catheter-related provoked UEDVT compared with patients with non-catheter-related provoked LEDVT (11). For this reason, clinicians must consider life-threatening PE due to delayed diagnosis and conservative management and should favor a more aggressive approach. Here, we present a case of subacute Paget–Schroetter syndrome complicated by pulmonary embolism and short-interval recurrent thrombosis.

## Case report

### Initial presentation

**Figure 2:**
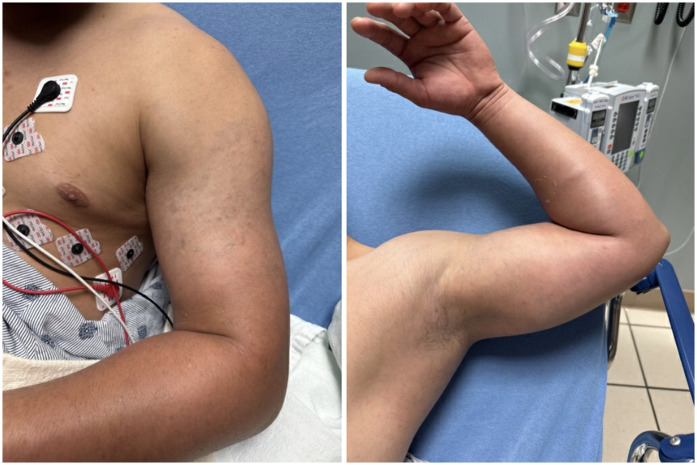
Swelling and discoloration of left upper extremity on initial presentation.

A 22-year-old physically fit male without prior medical problems presented to the emergency department (ED) complaining of pain, swelling, and discoloration of the left upper extremity, which had been developing over the previous two to three weeks (Figure 2).

He was an avid weightlifter, drove a delivery truck, and denied use of any supplements except creatine. He described the swelling as a gradual increase in size and pain without paresthesia. He complained of no motor deficits, and he denied any chest pain or shortness of breath. He also denied any traumatic injuries to the extremity, and he could not trace the pain to a particular workout. Upon further investigation, his mother reported having a history of lower extremity deep vein thrombosis after the birth of her youngest child, but there were no known hypercoagulable disorders in the family.

On exam, there was pronounced swelling to the left upper extremity. The unaffected right bicep measured 30 centimeters, while the left bicep was 38 centimeters in circumference. There was a mottled darkening of the skin over the bicep, resembling ecchymosis. Distal pulses were 2+ intact bilaterally, motor strength was 5+ bilaterally, and there was no paresthesia. Vital signs were: BP 131/92 mmHg, HR 72 bpm, RR 20/min, SpO2 100% on room air, T 36.9 degrees F.

Venous Doppler ultrasound of the left upper extremity was negative for thrombus, while CT of the chest with contrast revealed filling defects consistent with pulmonary emboli in the right pulmonary vasculature. A thrombus in the left subclavian vein was strongly suspected clinically but not adequately confirmed by the radiology report. Unfractionated heparin was initiated, and the patient was admitted for further evaluation.

### Admission

Due to high clinical suspicion for venous thromboembolism (VTE), repeat ultrasounds of all extremities were ordered. These were also negative for VTE. Clinical suspicion remained high, and Interventional Radiology (IR) was consulted to evaluate the patient for possible thrombectomy. Venogram demonstrated occlusive thrombus in the proximal-to-mid left subclavian vein, consistent with Paget–Schroetter syndrome. Thrombectomy and balloon angioplasty of the thrombus were performed and considered successful, demonstrating improved subclavian flow and decreased collateral flow. Additionally, a transthoracic echocardiogram (TTE) was ordered to assess for right-heart strain in the setting of pulmonary emboli; however, estimation of right ventricular systolic pressure could not be assessed.

A hypercoagulability workup was ordered during admission, including Factor V Leiden mutation, Prothrombin FII Mutation, beta 2-glycoprotein, antiphospholipid, and cardiolipin antibody, which were pending at discharge. Apixaban was initiated prior to release, with outpatient IR follow-up. When the patient reported to the primary care office for follow-up two weeks after discharge, he reported that his symptoms had worsened. It was noted that his left arm was still enlarged compared to the right and remained mottled in appearance. He was advised to return to the ED for further evaluation.

### Recurrence and clot extension

**Figure 3:**
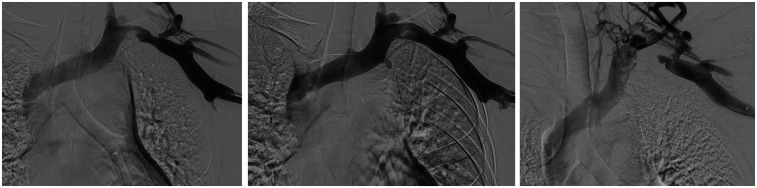
Left to right: initial venogram of LUE thrombosis, restored flow after CDT and recurrent thrombosis less than two weeks later.

CT imaging revealed a severe narrowing in the left subclavian vein, measuring approximately 2.5 cm in length, as the vein passed between the first rib and clavicle (Figure 3).

Repeat venogram with angioplasty was performed but deemed ineffective. It was recommended that he undergo surgical decompression. Vascular surgery was consulted and referred the patient to an outside facility for further evaluation. The patient was discharged pending this referral. However, on follow-up at the primary care office two days later, he reported worsened pain and discoloration of the left upper extremity. He was instructed to return to the ED. Complete occlusion of the left subclavian vein was again revealed on repeat CT scan. Ultrasound demonstrated thrombus within the left subclavian vein extending into the axillary, proximal cephalic, and throughout the brachial and basilic veins. Transfer to a quaternary facility was expedited.

### Surgical intervention

At the transfer facility, the patient underwent venous thoracic decompression of the left subclavian vein via left first rib resection and anterior and middle scalenectomy. The anterior scalene was divided from its insertion, mobilized medially, and completely resected from its origin at the transverse process of the cervical vertebra. The brachial plexus was lifted, and the middle scalene was resected from the origin to avoid sensation to the first rib. Inflammation was noted, along with venous collaterals that caused adhesions, necessitating the removal of the muscles in a piecemeal manner. The first rib was dissected from its origin to the insertion and also removed piecemeal, dissecting from the intercostal muscle and the intact pleura. The wound was irrigated, hemostasis obtained, a Jackson-Pratt drain secured, and closure was accomplished in multiple layers.

The patient was monitored in the Vascular Intensive Care Unit. Subsequently, he was deemed stable on post-operative day three and discharged home. When following up with his primary care provider, he reported significant improvement in his symptoms. Swelling and discoloration had largely resolved. At the time of writing, the patient had negative cardiolipin, beta 2-glycoprotein, antiphospholipid, and Factor V Leiden levels, while AT III, proteins C and S remain pending (Table 1).

**Table 1:** Timeline of clinical events from presentation to surgical intervention.

Relevant past medical history and interventions			
22-year-old physically fit male with no past medical or surgical history, not on any medications. Weightlifter, delivery driver, creatine supplements. No family history of hypercoagulability disorder, mother reports history of deep vein thrombosis after birth of youngest child.			
Dates	Encounter summaries	Diagnostic testing	Interventions
Day 1 (6/26) (Presentation)	2–3 weeks gradual onset left upper extremity pain, swelling, discoloration, without motor deficits, chest pain, or shortness of breath.	Left upper extremity (LUE) Doppler ultrasound (US) negative, chest computed tomography (CT) showed right-sided filling defects consistent with pulmonary emboli.	Started on unfractionated heparin (UFH).
	Exam: vitals stable, left arm swelling (38 cm vs. 30 cm), mottling. Mother with postpartum deep vein thrombosis (DVT). Suspected left subclavian thrombus. Stable, admitted for evaluation.		
Day 2–3 (6/27–6/28)	Due to high suspicion of DVT, therapeutic UFH continued, repeat imaging ordered, and interventional radiology (IR) consulted: venogram and thrombectomy completed; successful. Hypercoagulability workup ordered. Echocardiogram ordered to evaluate for right heart strain. With improvement of symptoms post thrombectomy, apixaban was initiated, and patient discharged with outpatient IR follow-up.	Repeat extremity ultrasounds negative. IR venogram: occlusive thrombus in left subclavian vein. Ordered, pending: Factor V Leiden, Prothrombin FII, beta 2-glycoprotein, antiphospholipid, and cardiolipin antibodies.	Thrombectomy + balloon angioplasty (successful). Started apixaban.
Day 14–16 (7/8–7/10)	2 weeks later, primary care physician (PCP) noted persistent and worsened swelling, mottling of LUE while on anticoagulation without missed doses. Referred to the Emergency Department (ED). Imaging obtained. IR consulted: venogram severe narrowing, angioplasty unsuccessful. Recommended surgical decompression, vascular surgery consulted. Referred to outside facility for surgical decompression. Discharged pending referral.	CT and venogram: severe narrowing in left subclavian vein 2.5 cm in length at costoclavicular junction.	Repeat angioplasty unsuccessful. Surgery recommended.
Day 18 (7/12)	2 days later, PCP follow-up with worsening pain and discoloration. Referred back to the ED. Imaging obtained, extension of thrombus noted. Expedited transfer to quaternary center for decompressive surgery.	CT: complete occlusion of left subclavian vein.	Transfer to a higher level of care for expedited surgery.
		US: thrombus extending into axillary, cephalic, brachial, basilic veins.	
Day 18–21 (7/12–7/15)	At accepting quaternary center, surgical venous thoracic decompression achieved, adhesions/venous collaterals noted. Admitted to vascular intensive care unit (VICU), stable for discharge at 3 days postoperative.	Hypercoagulability workup:	First rib resection with anterior and middle scalenectomy, followed by placement of Jackson-Pratt drain and 3-day post-operative VICU stay. Stable.
		Negative: cardiolipin, beta-2-glycoprotein, antiphospholipid, and Factor V Leiden.	
		Pending: AT III, proteins C and S.	
	At PCP follow-up: swelling/discoloration resolved, significant symptom improvement noted. Hypercoagulability workup: negative at this writing.		

## Discussion

Paget–Schroetter syndrome (PSS) is a rare venous manifestation of thoracic outlet syndrome. It occurs in only 0.5–1 in 100,000 cases, with a predilection for young, otherwise healthy males (4). It is often referred to as “effort thrombosis” due to the pathophysiology behind clot formation. While hypercoagulable states must always remain on the differential, a thorough evaluation of the history and physical components will likely point to a diagnosis of Paget–Schroetter syndrome. Young, adult male athletes, especially those who perform repetitive overhead motions, such as baseball, swimming, or weightlifting, should raise a red flag during the initial evaluation (9). Occupations involving continuous overhead work, such as electricians, plumbers, and mechanics, are also at high risk (9).

For those presenting with suspected PSS, a chest x-ray to rule out the existence of a cervical rib and a point-of-care ultrasound to evaluate Doppler flow are useful tools in the emergency department for initial diagnosis (12). Doppler compression ultrasonography remains the most efficient test for detecting subclavian thrombi, while MRI is the most accurate (13). Consideration for the development of pulmonary embolism (PE) in the setting of suspected PSS is paramount. While there has been some debate over the incidence of PE in upper extremity deep vein thrombosis (UEDVT) vs. lower extremity deep vein thrombosis (LEDVT), a recent critical care study found no significant difference between the two locations in the formation of PE (10). Disorders of coagulation should be investigated prior to heparin initiation to prevent inconclusive results. ED clinicians can assist in the overall treatment plan by ordering cardiolipin, beta-2 glycoprotein, antiphospholipid, proteins C and S, as well as Factor V Leiden tests, prior to initiating anticoagulant therapy. Otherwise, it is recommended that these tests be performed within two weeks after discontinuation of anticoagulant therapy, with exact timing specific to the type of anticoagulant employed (6).

Although there is no current consensus, treatment for PSS initially follows a conservative approach (CDT with anticoagulation), as over half of cases resolve without surgical intervention (14). However, resolution of the presenting thrombus does not necessarily prevent recurrence where anatomy has predisposed certain individuals, and long-term complications may include disability or even death related to post-thrombotic syndrome, recurrent thrombosis, or pulmonary embolism. A recent meta-analysis found that 46% of those treated with anticoagulation alone have persistent symptoms (14). Chronic recurrence often leads to vascular scarring and obliteration of the vessel, rarely amenable to surgical correction (15).

Given an acute phase, primary thrombosis, anticoagulation may be sufficient to treat the condition. True primary thrombosis is rare, and many primary diagnoses are amended as CCJ compression, a hypercoagulable disorder, or malignancy is found. In PSS, there is greater efficacy with CDT and subsequent first rib resection and scalenectomy, which has been shown to prevent recurrence in 96% of cases (14). While conservative therapy (anticoagulation + CDT) may be an effective treatment at almost 100% efficacy for clots of only a few days of age, thrombosis has been shown to recur in 46% of cases with anatomic extrinsic compression, potentially leading to chronic fibrosis (6).

In young, healthy patients with relatively few surgical risks, prompt surgical correction of the anatomy following revascularization has been effective in preventing restenosis and the development of post-thrombotic syndrome (15). It has been recognized and further recommended by some that rib resection may be overtreating the thrombosis, even if a subclavian lesion is identified (6). However, it has also been shown that >40% of conservatively treated patients ultimately require rib resection for repeat thrombosis, suggesting an aggressive approach may be more efficient and even safer (6).

In previously published trials, where immediate first rib resection and anticoagulation therapy follow CDT, 100% effectiveness was reported (6). In a recent systematic review and meta-analysis, a subgroup of 1,309 patients from 20 studies found significant improvement in venous patency and symptom resolution in patients who underwent first rib resection with or without venoplasty (16).

Given the favorable outlook for a proactive approach to treatment, even in acute phase UEDVTs, we believe it is reasonable to take a more aggressive approach to subacute phase thrombi as well. Subacute phase thrombi are usually harder to treat as the clot is increasingly organized and therefore more resistant to thrombolysis (6). As such, the more effective methods of disrupting the thrombus, including pharmacomechanical thrombectomy and mechanical thrombectomy (both with 100% efficacy), are often employed (17). Davies et al, propose an algorithm to approach the management of UEDVT, suggesting first rib resection if evidence of compression is noted (17).

While first rib resection is not typically pursued on initial discovery of PSS, given the extremely fast window of recurrence and extension of the clot at two weeks post mechanical thrombectomy and anticoagulation, it is likely our patient would have benefited from immediate first rib resection. He was from a non-native speaking demographic, which made him at high risk for loss to follow-up. Additionally, he was a young, athletic overhead amateur athlete, which would have placed him in a demographic highly suspicious for CCJ compression even without venographic evidence of compression. Therefore, we support an aggressive approach to offering definitive treatment for PSS, considering the very small likelihood of overtreating patients in this regard, even in the subacute phase of presentation.

## Conclusion

Effort thrombosis, although relatively rare, is likely to be encountered by most hospital-based physicians during their careers. Efficient and thorough diagnosis involves a chest x-ray to rule out a cervical rib, a point-of-care ultrasound to locate the thrombus, and formal studies, along with an early investigation into hypercoagulability profiles prior to initiating anticoagulant therapy. Careful consideration to rule out concurrent pulmonary embolism is critical, as initially asymptomatic patients can develop clinically significant PE as emboli migrate. The risk of post-thrombotic syndrome disability, recurrent clot formation, and life-threatening emboli must be weighed in the balance when considering conservative management. Aggressive therapy with definitive treatment should be employed when benefits outweigh surgical risks, with decompression occurring swiftly following clot destruction.

## Data Availability

The original contributions presented in the study are included in the article/Supplementary Material, further inquiries can be directed to the corresponding author.
